# Correlation between Depth of the Curve of Spee and Some Specific Malocclusion Characteristics in a Population from Sibiu County, Romania—A Cross-Sectional Study

**DOI:** 10.3390/jcm13102750

**Published:** 2024-05-07

**Authors:** Vasile Calin Arcas, Ioan Andrei Tig, Doru Florian Cornel Moga, Alexandra Lavinia Vlad, Adriana Saceleanu, Anca Maria Fratila

**Affiliations:** 1 Doctoral School of Biomedical Sciences, Faculty of Medicine, Lucian Blaga University of Sibiu, 550169 Sibiu, Romania; calin.arcas@ulbsibiu.ro; 2 Department of Dental Medicine, Faculty of Medicine and Pharmacy, University of Oradea, 410028 Oradea, Romania; 3 Department of Dental Medicine and Nursing, Faculty of Medicine, Lucian Blaga University of Sibiu, 550169 Sibiu, Romania; adriana.saceleanu@ulbsibiu.ro (A.S.); anca.fratila@ulbsibiu.ro (A.M.F.); 4 Military Clinical Emergency Hospital, 550024 Sibiu, Romania; 5 Doctoral School of Biomedical Sciences, Department of Dental Medicine, Faculty of Medicine and Pharmacy, University of Oradea, 410087 Oradea, Romania; alvlad@uoradea.ro

**Keywords:** Curve of Spee, overjet, overbite, malocclusion

## Abstract

**Background:** This study aimed to investigate the relationship between the Curve of Spee (COS) depth and malocclusion characteristics in a population from Sibiu County, Romania. The research sought to understand how the COS’s anatomical dimensions correlate with different classes of malocclusion and the position of the frontal teeth. **Methods:** A total of 265 participants from the Dentistry Ambulatory of the Military Hospital of Sibiu were included in this study. It employed digital intraoral scanning technology to measure the COS, overjet, and overbite, with malocclusion classification. Statistical analyses, including correlation and regression, were conducted to examine the relationships between COS depth, frontal teeth position, and malocclusion characteristics. **Results:** The average COS depth measured was 1.0564 mm, presenting variability when compared to existing literature. Most of the study participants had Class I malocclusion. A significant positive correlation between COS depth and overjet was identified, with no notable gender-based differences in these occlusal parameters. **Conclusions:** The findings affirm the integral role of COS in occlusal dynamics and malocclusion diagnosis. The COS measurement of a mean of 1.0564 mm, when compared to other results emphasizes the differences in occlusal curvature among various populations. The results contribute to a refined understanding of occlusal relationships, supporting the need for personalized orthodontic and prosthetic treatments based on precise anatomical measurements.

## 1. Introduction

The Curve of Spee (COS) is a fundamental concept in dentistry, specifically important in orthodontics and prosthetic dentistry [1]. It was first described by the German anatomist F. Graff Von Spee in 1890 [2]. The curve, which is perceived in the sagittal plane, represents the natural alignment of the teeth in the lower and upper jaws [3]. COS plays a key role in the biomechanics of mastication, influencing the ratio of crushing to tearing pressures between the back teeth and the effectiveness of biting forces during mastication [3,4]. It has been proposed that this curve improves the efficiency of food processing by increasing the mechanical advantages of the masticatory system [5].

The COS impacts the equilibrium between crushing and tearing pressures in the mouth and improves the effectiveness of biting forces during mastication, hence boosting the mechanical benefits of the masticatory system [4].

From an orthodontic perspective, the depth of the COS plays a crucial role in the diagnosis and treatment of dental misalignments [6]. It is typically more noticeable in Class II malocclusions and less prominent in Class III cases [7]. Treating these misalignments typically requires adjusting the sagittal curve to establish a harmonious occlusal plane [8]. This is often accomplished by employing techniques such as anterior intrusion or posterior extrusion [9].

The COS plays a vital role in the field of restorative dentistry by guiding the design of dental restorations and prostheses [10]. It is essential to accurately replicate the natural occlusal curve in order to achieve optimal functional efficiency and aesthetic balance [11]. To adequately simulate the natural alignment of the teeth, it is necessary to accurately replicate this shape in comprehensive dental restorations [10,11,12].

Recent investigations have confirmed the significance of the COS in the planning of orthodontic and prosthetic treatments. The research undertaken by Jan et al. (2020) [13] and investigations carried out at the M.R. Ambedkar Dental College and Hospital, Bangalore have yielded significant findings regarding the relationship between the COS and certain dental and skeletal factors. These studies have emphasized the need to consider the COS when planning full therapy in order to improve occlusal functionality and achieve better cosmetic outcomes.

Furthermore, the COS is inherently connected to the measurement of overjet and overbite in the diagnosis and treatment of malocclusion [14]. Studies have shown that there is a direct relationship between the depth of the COS and an increased horizontal overlap of the upper and lower teeth (overjet) [15] as well as an increased vertical overlap of the front teeth (overbite), especially in cases where there is a Class II malocclusion [5,16]. The depth of the COS directly impacts the horizontal and vertical overlapping of the teeth. The presence of these abnormalities requires a thorough comprehension of the biomechanical and functional importance of the occlusion plan for formulating suitable treatment strategies [17].

To comprehend the impact of the depth of the COS on dental alignment, one must consider several elements, including the relationships between the anterior and posterior teeth [18], the angle at which the incisors are positioned [19], the patterns of vertical tooth eruption [20], and the functional alignment of the teeth during mastication [21]. These aspects illustrate how a pronounced COS can exacerbate malocclusions, affect tooth orientation, and influence vertical and horizontal teeth relationships [22], ultimately guiding the strategic approaches in orthodontic and restorative dental treatments [23].

In the etiology of malocclusion, overjet, and overbite are related to the depth of the COS, but the mechanism is more complex [24], as it involves a combination of genetic, environmental, and functional factors [25]. Irregularities in the sagittal occlusion curve can result in abnormalities in the way the teeth fit together, requiring orthodontic treatment to correct the alignment and restore proper tooth contact [26,27].

This article aims to investigate the relationship between the depth of the COS and other features associated with malocclusion, specifically focusing on overjet, overbite, and the class of malocclusion. The objective of this work is to measure the correlation between the anatomical characteristics of the COS and the extent and nature of malocclusion, thereby offering valuable insights into the mutual influence of these dental structures. The main distinction of our study is the use of digital intraoral scanning technology, which offers more precise and efficient measurements compared to traditional methods involving dental casts and manual measurements when compared with previous studies. The research was carried out on a representative group of individuals from Sibiu County, Romania, in order to ascertain the frequency and influence of changes in COS depth on malocclusion.

## 2. Materials and Methods

This observational cross-sectional research was conducted by a team of researchers from “Lucian Blaga” University, in partnership with the Faculty of Medicine and Pharmacy within the University of Oradea and with the contribution of the Military Hospital of Sibiu’s Dentistry Ambulatory, all the 3 mentioned institutions being located in Romania. The study had a sample of 265 individuals, selected from patients in the Dentistry Ambulatory of the Military Hospital of Sibiu, Sibiu County, Romania.

The study population’s characteristics were thoroughly recorded, with a specific emphasis on age, gender, ethnicity, and overall health status. Every participant gave written informed consent, following a protocol that detailed the study’s extent, possible hazards, and advantages, with a focus on voluntary involvement and data privacy.

In order to establish the study’s legitimacy and compliance with ethical standards, the research protocol obtained clearance from the ethics commission of the Military Hospital of Sibiu. The data presented in this study are available on request from the corresponding author due to privacy and confidentiality considerations associated with conducting research within a military institution. The study involved some patients who are military personnel, necessitating strict adherence to the privacy and security protocols mandated by the Romanian Ministry of Defense.

The population included in this study was diverse and provided a suitable sample for investigating the relationship between COS, overjet, overbite, and the class of malocclusion. This allowed for a thorough and comprehensive analysis of the occlusal characteristics in the region.

The inclusion criteria stipulated that participants must not have undergone orthodontic treatment in the past, should have at most one tooth missing in each dental arch, and should not have any craniofacial deformities. When the first molar was missing or had significant restorative work, determining malocclusion class was achieved by evaluating the canine relationship, using the second molars as reference points, or assessing incisal relationships. The exclusion criteria included individuals with partial or complete tooth loss, detachable dentures or dental implants, head or neck tumors, mandibular fractures, or previous orthodontic treatments or orthognathic surgery. To assess the implementation of these criteria, all the individuals were analyzed by 2 separate physicians, all disagreements being solved by discussion and mutual agreement. 

The study employed a methodological approach that included recording digital intraoral scans of all subjects, followed by analysis using Exocad DentalCAD software V 3.2 (Exocad GmbH, Darmstadt, Germany) [28]. The measurements included the COS, which was quantified in millimeters and indicated whether it was concave, by a positive value of the measurement, or convex, by a negative value of the measurement, as well as the dimensions of overbite and overjet. Furthermore, the classification of malocclusion was conducted based on well-established orthodontic concepts [29]. 

The scanning was conducted utilizing a Helios 500 intraoral scanner, developed by Eighteeth, a division of Changzhou Sifary Technology Co., Ltd. (Sifary Medical, Changzhou, China), following the steps in the agreed protocol. The process involves a series of steps, including calibration, patient preparation, scanning, and data capture and quality check [30].

**Figure 1:**
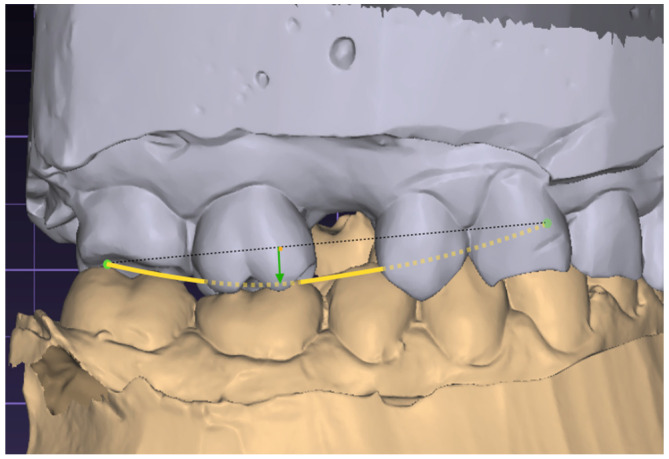
Measurement of the Curve of Spee.

To measure the COS, the operator locates the buccal cusp tips of the premolars and molars, as well as the incisal edge of the canine on one side of the mandibular arch. In Exocad V 3.2, a tool called a Curve or Arc Measuring Tool is utilized (Figure 1), to create a line that connects these points, so visually establishing the COS. The software determines the depth by measuring the perpendicular distance from the arc to the buccal cusp point of the first molar and then saves this measurement. The measurement was performed on both sides and the mean of the measurements was calculated for each patient. 

**Figure 2:**
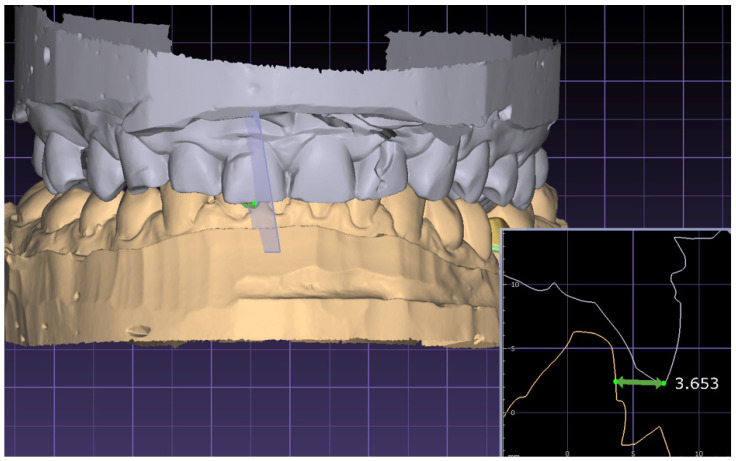
Measurement of the overjet.

The overjet measurement (Figure 2) involves identifying the external surface of the biting edges of the upper and lower front teeth. A line that is parallel to the occlusal plane is created by using the linear measurement tool to draw a line from the incisal edge of the mandibular incisor to the labial surface of the maxillary incisor. The length of this line, which represents the overjet, is measured and recorded. The overjet measurements vary, showing positive values when an overjet is present and negative values in cases of a reverse jet. In cases of an open bite, the measurement is done by assessing the value between the 2 parallel vertical lines that pass through the incisal edge of the upper tooth and the labial face of the lower one.

**Figure 3:**
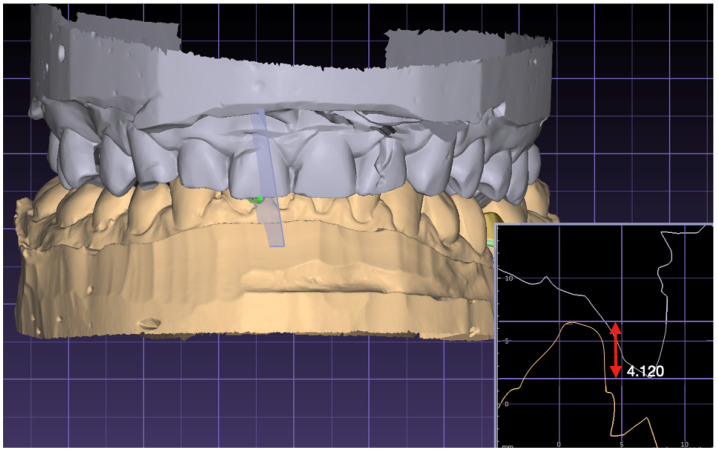
Measurement of the overbite.

To evaluate the overbite (Figure 3), the perspective is adjusted to provide the side view of the incisors. To measure the vertical overlap of the maxillary and mandibular incisors, a perpendicular line is drawn from the incisal edge of the upper incisor to the incisal edge of the lower incisor. This line must be at a ninety-degree angle to the occlusal plane. Exocad V3.2 has the capability to measure the overbite and subsequently records this measurement. The overbite values range from positive, indicating the presence of an overbite, to negative, which occurs in cases of an inverted occlusion or a negative sagittal step.

Following the measurement process, the initial data were thoroughly reviewed and verified, and it was made sure that the values were transferred correctly to the database, in order to ensure precision, and any necessary modifications are implemented. The measurements are stored in the patient’s digital file within Exocad v3.2 for the purpose of documentation and future utilization. 

**Figure 4:**
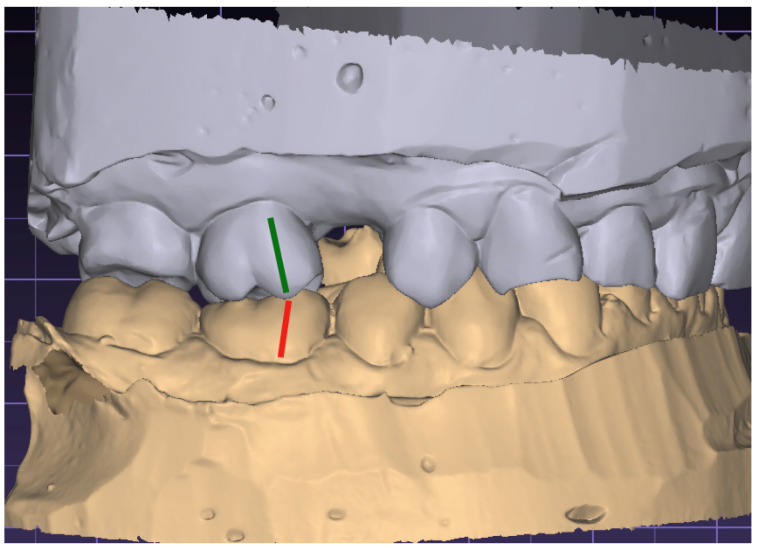
Evaluation of the malocclusion class (red line—the center of the labio-mesial cusp of first lower molar, green line—the center of the labio-mesial cusp of first upper molar).

The molar relationship is assessed by identifying the first molars in both arches and examining the relationship between the mesial-buccal cusp of the upper first molar and the buccal groove of the lower first molar. This helps determine if the malocclusion is classified as Class I, II, or III. The observation was made on both sides separately, as shown in Figure 4.

The process of documenting important observations was carried out by capturing screenshots or utilizing Exocad’s reporting features. These observations were subsequently compared to predetermined criteria for categorizing malocclusion, which is based on categorization of the angle.

The research utilized descriptive statistics to summarize both continuous and categorical data for statistical analysis. The SPSS version 24.0 for Windows software [31] was used for this purpose. The comparative analyses utilized Student’s *t*-test, chi-square test, Fisher’s exact test, and the Mann–Whitney test [32]. In addition, a multivariate logistic regression analysis was conducted, taking into account age and sex, to determine the independent relationships between depth of COS and overjet, overbite, and the malocclusion class. The threshold for statistical significance was established at a *p*-value below 0.05 [33].

This methodology intends to thoroughly investigate the relationship between the COS, overjet, overbite, and malocclusion class. Its purpose is to guide future dental treatment methods specifically for the population of Sibiu County, Romania.

## 3. Results

This study evaluated 265 individuals, ranging in age from 18 to 65 years, with a mean age of 35 years. The study analyzed the depth of the COS, as well as the measurements of overjet, overbite, and malocclusion class. The demographic analysis revealed that out of the total participants, 175 were females, accounting for 66.04% of the sample, while 90 were males, making up 33.96% of the sample. This indicates a greater proportion of female participants in the study.

The following table (Table 1) summarizes the population characteristics of the observational cross-sectional study participants. This gives an overview of the demographic makeup of the study population.

Most participants in the study displayed satisfactory oral hygiene, with 56.60% reporting good hygiene despite various dental issues: 31.69% had tooth decay, 9.43% faced gum disorders, and 97.16% had received prior dental treatments. Although most had a complete set of permanent teeth, some had missing teeth due to extractions. The study encompassed a demographically diverse group from Sibiu County, including various professional and economic backgrounds, with educational levels ranging from primary (13.21%) to higher education (37.74%). Ethnically, the majority (90.57%) were Romanian, reflecting the local demographic, which supported a broad understanding of occlusal variations in a multi-ethnic cohort.

The following table (Table 2) encapsulates the key characteristics of the study population, providing a comprehensive view of their oral health status, dental history, demographic makeup, and educational levels.

The average depth of the COS was 1.0564 mm, with a standard deviation of 1.21667. The depth ranged from −2.10 mm to 5.20 mm. The median values for the left and right sides were 0.950 mm and 1.173 mm, respectively, as shown in Table 3. The average measures for overjet and overbite were 2.80 mm (standard deviation 2.89) and 1.22 mm (standard deviation 0.99), respectively (Table 4).

**Figure 5:**
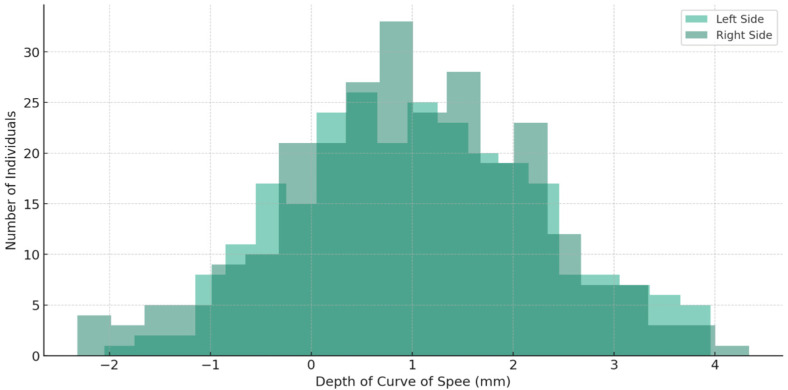
The distribution of the depth of COS for both the left and right sides among 265 individuals.

**Figure 6:**
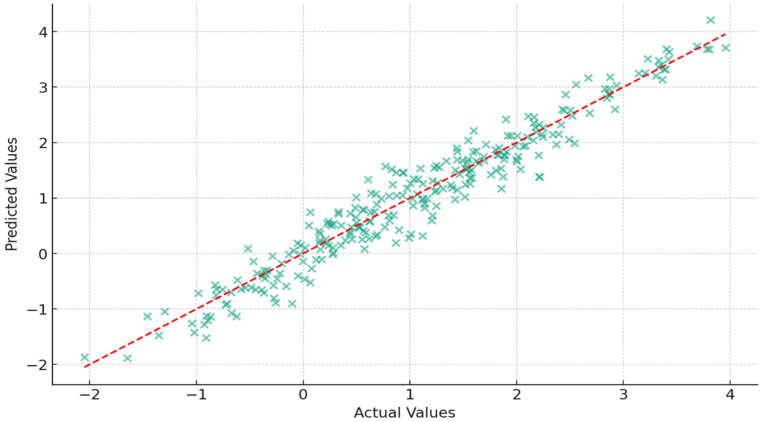
The predicted versus actual values for the depth of the COS. Each point represents a patient, with their actual measurement value on the x-axis and the predicted value on the y-axis. The red dashed line indicates a perfect prediction where the predicted values would equal the actual values. The scatter of points around this line illustrates the variation between the predicted and actual measurements.

**Figure 7:**
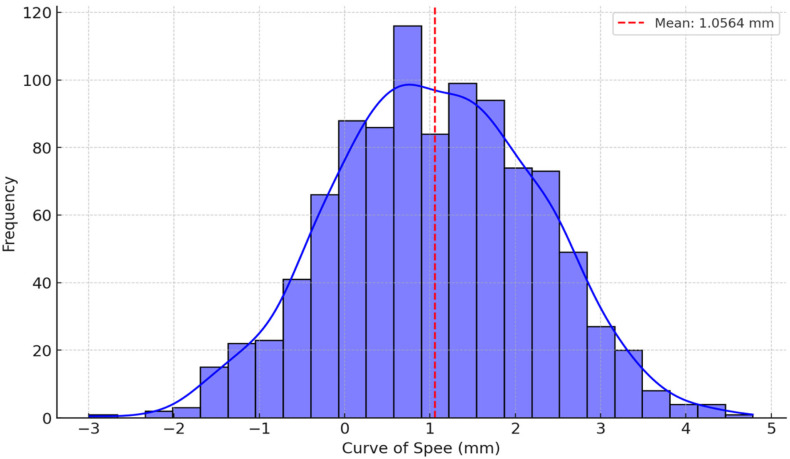
The normal distribution of COS depth values.

**Figure 8:**
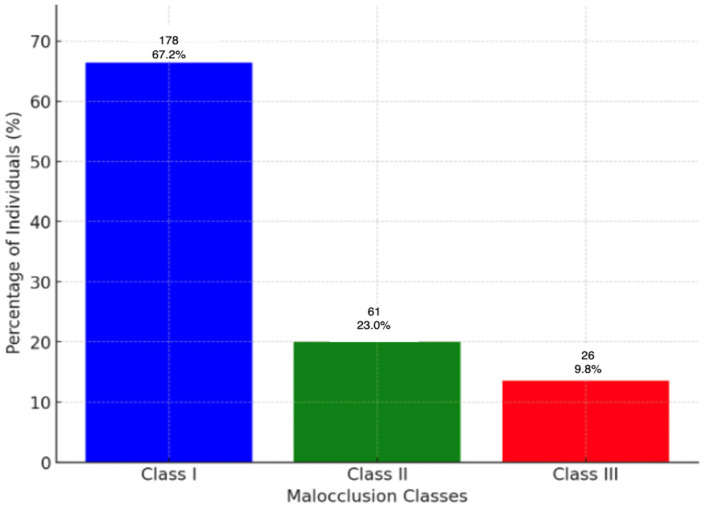
The distribution of malocclusion classes.

The values extracted are represented in the following figures (Figure 5, Figure 6, Figure 7 and Figure 8).

The majority of the population (almost two-thirds) had Class I malocclusion, with Classes II and III being less common (Table 5). Class III malocclusion was substantially related to negative values for overjet and overbite, while overjet values over 3 mm were indicative of Class II malocclusion.

**Figure 9:**
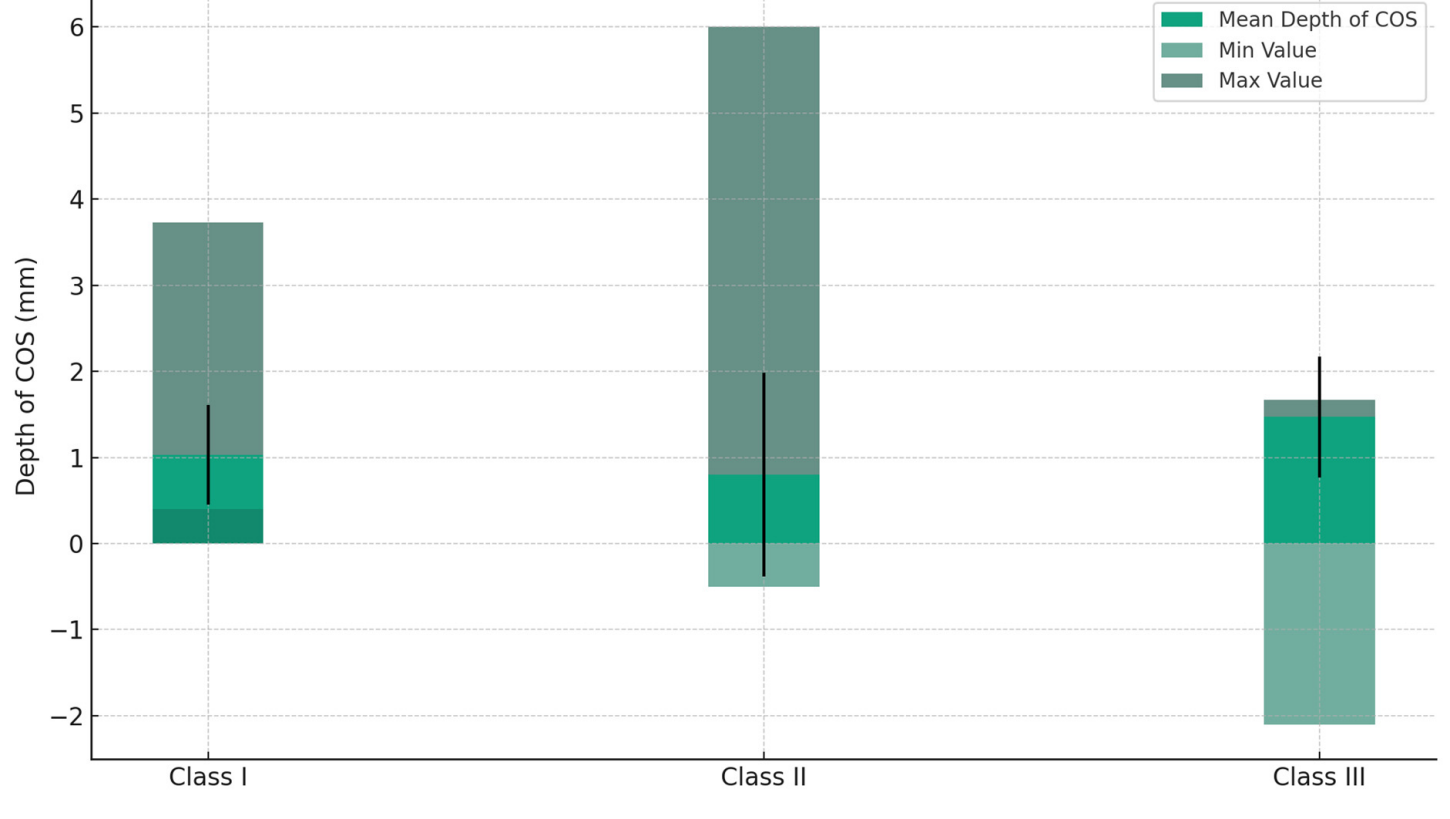
The depth of the COS across different malocclusion classes, showcasing the mean depth, standard deviation (indicated by error bars), minimum, and maximum values for each class.

The use of statistical analysis utilizing SPSS software V 24 unveiled intricate connections between the observed characteristics and demographic parameters. The average depth of the COS exhibited small fluctuations among various malocclusion classes as shown in Table 6 and Figure 9, with Class I malocclusion displaying the most consistent average values, suggesting a rather steady COS in this category. Class II and III malocclusions had greater variability, indicating that these classes may be more prone to extreme variations in the curvature of the occlusal plane.

The measurements of overjet and overbite exhibited a complex relationship, with a higher overjet commonly indicating Class II malocclusion, while negative values were more indicative of Class III cases. The statistical tests demonstrated these links, with the chi-square and Kruskal–Wallis tests proving the importance of these relationships (Table 7).

The statistical analysis (Table 8) showed a significant positive correlation (r = 0.715, *p* < 0.01) between the depth of the COS and overjet, as well as a negative correlation (r = −0.598, *p* < 0.01) between overbite and overjet. The study observed a negative correlation (r = −0.0197, *p* < 0.01) between the depth of the COS and overbite, with overjet working as a mediator, strongly suggesting that as the curvature of the occlusal plane increases, there is a tendency for the overjet to increase as well, which is particularly evident in Class II malocclusion. 

**Figure 10:**
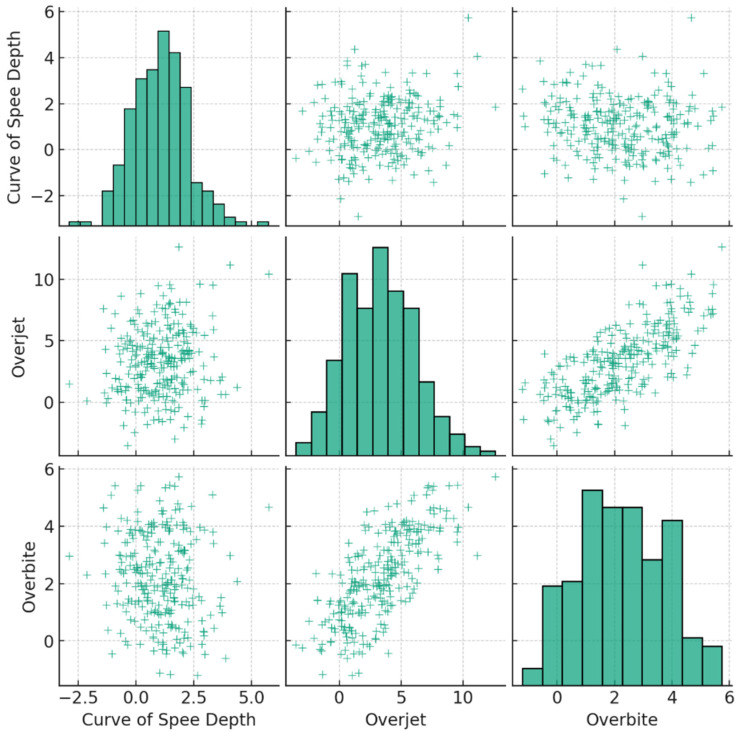
The correlation between studied parameters: the depth of COS, overjet, and overbite. Each scatter plot illustrates the relationship between two parameters, with the trend line indicating the direction and strength of the correlation. Histograms on the diagonal provide the distribution of each variable. This visualization helps to understand the interplay between these critical measurements.

The depth of the COS, overjet, overbite, and malocclusion class were analyzed using statistical methods (Figure 10). Continuous factors were analyzed using Student’s *t*-test, while categorical variables were analyzed using chi-square testing. The aim was to determine if there were any significant gender-related differences in these characteristics. However, the findings did not reveal any notable disparities depending on gender.

To be more precise: The depth of COS was compared between males and females using a Student’s *t*-test. The results indicated that there was no significant variation in the mean depth of COS between the two genders. This suggests that the curve of the occlusal plane is consistent regardless of gender.

The examination of overjet measurements also revealed no significant variations based on gender. Males and females displayed a wide range of overjet values, with no statistically significant disparity in their average measures.

The investigation of overbite revealed that there is no significant difference between boys and girls. This conclusion is based on the findings of the Student’s *t*-test.

A chi-square test was conducted to assess the difference of malocclusion classes (Class I, II, III) between genders. The results showed (Table 9) that there was no significant difference in malocclusion class distribution between males and females. There was an equal probability for both genders to exhibit any of the three categories of malocclusion.

Overall, this investigation confirmed the differences in the COS, overjet, and overbite among various malocclusion classes, highlighting significant relationships that could have implications for restorative or orthodontic diagnosis and treatment planning. 

## 4. Discussion

A complete understanding of dental diagnosis and therapeutic procedures relies heavily on knowing the complex correlation between the depth of the COS and several occlusal characteristics. The depth of this curve is not just determined by the individual’s occlusal morphology, but it also plays a vital role in the overall functionality of the masticatory system.

This study‘s results align with earlier observations in the scientific literature. The analysis indicated that the depth of the COS differed depending on the class of malocclusion. Class I malocclusion exhibited the highest level of consistency in COS depth, which is consistent with the conventional gnathological belief that Class I malocclusion indicates a more harmonious occlusal relationship. This was confirmed in other studies such as Emrich et al. (1965) [29]. 

The examination of overjet and overbite revealed a complex and nuanced depiction. As anticipated, Class II Division 1 malocclusion often presents with a greater overjet, characterized by the maxilla being positioned anteriorly in relation to the mandible. In contrast, Class II Division 2 malocclusion does not typically show a greater overjet. In both divisions of Class II malocclusions, the mandible is generally positioned posteriorly relative to the maxilla, affecting the dental and occlusal dynamics in combination [34], as demonstrated in the investigations conducted by Baydas et al. [35] and Shashidhar et al. [36]. These issues can result in reduced efficiency, greater tooth wear, and aesthetic problems, emphasizing the need for orthodontic treatment to correct the curve and restore proper alignment of the teeth [37].

In contrast, the presence of negative values in overjet and overbite measurements was primarily observed in Class III instances, characterized by a prognathic jaw or a mandible positioned anteriorly to the maxilla, retrognathic or small maxilla, or a combination of those factors [38]. These findings align with the conventional classifications and clinical manifestations of these malocclusion categories.

The correlation between overbite and overjet, and their combined influence on the curvature of the occlusal plane, as measured by the COS, highlights the intricate nature of occlusal dynamics [24,39].

The curvature of the jaw helps with efficient mastication by conforming to the optimal shape of the arch, which is necessary for equally distributing the forces of biting and ensuring a stable function of the temporomandibular joint [40].

Leveling the COS in orthodontic treatment is not solely for aesthetic purposes but is essential for attaining functional occlusal connections [41]. The studies conducted by Krüsi et al. [15] and Jan et al. [13] have demonstrated that there is a correlation between the depth of the curve and the craniofacial morphology. This correlation is important in order to develop treatment plans that are complete and aligned with the patient’s overall facial structure.

The research conducted by Schieffer et al. [16] demonstrates the importance of regulating COS in prosthetic rehabilitations, particularly in cases that involve long-span restorations. This is achieved through a multidisciplinary strategy that combines knowledge from prosthodontics and orthodontics. The form of the curve has a substantial impact on the outcome of the prosthetic, highlighting the need for careful planning and execution in dental restorations [42].

Studies have consistently demonstrated that the extent of the COS differs across various malocclusion classes. Paes-Souza et al. [43] and Yan A. et al. [44] observed significant disparities in the depth of the curve among Class I, II, and III malocclusions, with Class II malocclusions frequently exhibiting a greater depth. This discovery is consistent with the research conducted by Nouh et al. [45], which revealed that individuals with Class II malocclusion had a notably more pronounced COS in comparison to those with Class I malocclusion. This indicates a potential connection between the depth of the curve and the developmental cause of malocclusion.

The investigation by Alkhalaf et al. [40] also explored the aspect of sexual dimorphism regarding the COS. Their research indicated that the gender of the participants did not have a substantial effect on the depth of the COS, overjet, or overbite. The absence of sexual dimorphism suggests that differences in the depth of the curve are mostly influenced by individual anatomical and occlusal factors rather than gender-specific characteristics.

The reviewed studies consistently confirm that the depth of the COS has significant orthodontic effects. The research conducted by Krüsi et al. [15] and Jan et al. [13] has revealed a detailed understanding of the relationship between the curve and craniofacial patterns and cephalometric factors. This knowledge is crucial for orthodontic treatment planning and execution [46].

Additional research is required to clarify the developmental variables that influence the establishment of the COS and its variation among various races and age groups. Utilizing advanced imaging techniques and three-dimensional modeling can provide a more comprehensive understanding of how the curve and craniofacial growth patterns interact [47]. This can lead to more accurate predictions and personalized treatment.

In the current study, the mean depth of the COS was measured at 1.0564 mm, which presents an interesting comparison to previous research. For instance, the work by Steven D. Marshall et al. [48] in 2008 reported a mean COS depth of 1.92 mm, while another study by Krishnamurthy et al. [49] (2017) indicated a mean of 1.56 mm. These variations in COS depth measurements across different studies highlight the diversity of occlusal curvature present in various populations and underscore the influence of demographic, skeletal, and dental factors on the COS.

The depth of the occlusal plane is closely connected to both functional and aesthetic features, which have an impact on how malocclusion situations are treated [50,51,52,53]. Gaining a more profound comprehension of how the depth of the COS interacts with malocclusion classes, overjet, overbite, and craniofacial anatomy improves our capacity to anticipate outcomes and customize restoratives and orthodontic treatments. Furthermore, recognizing the lack of sexual dysmorphism in the depth of the curve supports the idea that evaluations and treatments should be tailored to each person’s unique anatomical and occlusal features, rather than being generalized based on gender. Further investigation is necessary to study these connections in order to enhance better treatments and satisfy patient outcomes.

## 5. Conclusions

This study underscores the significance of the COS as a vital anatomical feature influencing mastication mechanics and dental occlusion dynamics. The key finding was that the average depth of the COS was 1.0564 mm. This measurement, when compared to other results (1.92 mm and 1.56 mm) emphasizes the differences in occlusal curvature among various populations.

An important advancement of the study is the confirmation of digital scanning as a more dependable technique for assessing the COS. Digital scanning, in contrast to conventional approaches, offers computerized measurements that guarantee accuracy and minimize the chances of human mistakes, marking a notable breakthrough in dental diagnostics. Moreover, a significant proportion of the study participants had Class I malocclusion, consistent with previous studies, indicating that this is the prevailing kind of malocclusion. The consistency shown across several studies strengthens our understanding of the characteristics of Class I malocclusion, particularly its consistent depth of the COS, which plays a role in the stability and functionality of the occlusal plane. Additionally, this study found an important and positive correlation between the depth of the COS and overjet, providing further clarification on the intricate dynamics of occlusal interactions. Significantly, there were no apparent gender-related disparities found in the extent of the COS, overjet, or overbite, which supports the need for an individualized approach to dental care that goes beyond gender categorizations.

The observed variations in overjet and overbite among different classes of malocclusion are likely influenced by biological factors, functional considerations, and the specific characteristics of the study sample. These findings enhance our understanding of orthodontic classification and underscore the multifactorial nature of dental occlusion.

This study enhances the field of dentistry by offering a more comprehensive understanding of the COS’s function in occlusal alignment and by advocating for technological developments that improve patient results.

## Figures and Tables

**Table 1:** Study population characteristics.

**Headcount of individuals**							
265							
**Age (years)**							
Mean	SD		Median		Min		Max
35	12		35.55		18		65
**Gender**							
Males				Females			
90		33.96		175		66.04%	

**Table 2:** Key characteristics of the study population, providing a comprehensive view of their oral health status, dental history, demographic makeup, and educational levels.

**Hygiene status**		
Good hygiene	150	56.60%
Poor hygiene	115	43.40%
**Dental problems**		
Tooth decay	84	31.69%
Gum problems	25	9.43%
Previous dental treatments	231	97.16%
**Patients with missing teeth**		
Fewer than 1 teeth per arch	40	15.09%
**Ethnicity**		
Romanian	240	90.57%
Other	25	9.43%
**Level of Education**		
Primary education	35	13.21%
Secondary education	130	49.06%
Higher education	100	37.74%

**Table 3:** Descriptive values of COS Depth.

**Values of the depth of the Curve of Spee (mm)**									
Mean value		SD		Median		Min. value		Max. value	
1.0564		1.21667		1.0564		−2.10		5.20	
**Left side values**					**Right side values**				
Mean value	SD	Median	Min. value	Max. value	Mean value	SD	Median	Min. value	Max. value
0.982	1.593	0.950	−1.518	5.20	1.131	1.569	1.173	−2.10	3.756

**Table 4:** Descriptive values of COS, Overjet, and Overbite.

	Mean Value	SD	Median	Min. Value	Max. Value
Mean of COS (mm)	1.0564	1.21667	1.0564	−2.10	5.20
Overjet (mm)	2.80	2.89	2.81	−1.82	6.79
Overbite (mm)	1.22	0.99	1.46	−0.92	2.77

**Table 5:** Distribution of Malocclusion Classes.

Class of Malocclusion					
Class I		Class II		Class III	
N	Percent	N	Percent	N	Percent
178	67.2%	61	23.0%	26	9.8%

**Table 6:** A structured overview of COS depth across the different classes of malocclusion, highlighting the consistency and variation within each group.

Malocclusion Class	Mean Depth of COS (mm)	Standard Deviation	Min Value	Max Value
Class I	1.03	0.58	0.4	2.7
Class II	0.8	1.18	−0.50	5.2
Class III	1.47	0.70	−2.10	0.2

**Table 7:** The relationship between overjet, overbite, and malocclusion classes, using chi-square tests reflecting the complex interplay and statistical significance of these associations.

Malocclusion Class	Overjet Range (mm)	Overbite Range (mm)	Significance (*p*-Value)
Class I	−1.82 to 3.00	−0.92 to 2.77	>0.05
Class II	>3 to 6.79	0 to 3	<0.01
Class III	<−1.82	<−0.92	<0.01

**Table 8:** Correlation between observed parameters.

Parameters	Correlation Coefficient (r)	*p*-Value
COS Depth and Overjet	0.715	<0.01
Overbite and Overjet	−0.598	<0.01
COS Depth and Overbite	−0.197	<0.01

**Table 9:** Gender-based analysis of analyzed parameters.

Parameter	Gender	Mean	Standard Deviation	Min Value	Max Value	*p*-Value (Student’s *t*-Test/Chi-Square)
COS Depth	Male	1.03	0.50	−2.10	5.20	0.45
	Female	1.08	0.52	−2.00	5.10	
Overjet	Male	2.80	2.89	−1.82	6.79	0.62
	Female	2.81	2.87	−1.80	6.77	
Overbite	Male	1.22	0.99	−0.92	2.77	0.57
	Female	1.23	1.00	−0.90	2.75	
Malocclusion Class	-	-	-	-	-	0.53 (Chi-square)

## Data Availability

The data presented in this study are available on request from the corresponding author due to privacy and confidentiality considerations associated with conducting research within a military institution. The study involved patients who are military personnel, necessitating strict adherence to the privacy and security protocols mandated by the Ministry of Defense.
